# Pediatric End-of-Life Simulation Workshop to Clinical Care: Lasting Implications on Clinical Practice

**DOI:** 10.1089/pmr.2023.0065

**Published:** 2024-03-28

**Authors:** Kayla Solstad, Heidi Kamrath, Sonja Meiers, Naomi Goloff, Johannah M. Scheurer

**Affiliations:** ^1^ Department of Pediatrics, University of Minnesota Medical School, Minneapolis, Minnesota, USA.; ^2^ Neonatology, Children's Minnesota, Saint Paul, Minnesota, USA.; ^3^ Department of Nursing, University of Wisconsin-Eau Claire, Eau Claire, Wisconsin, USA.; ^4^ Department of Pediatrics, McGill University, Montreal, Quebec, Canada.; ^5^ Department of Pediatrics, Faculty of Medicine and Health Sciences, Institute of Health Sciences Education, McGill University, Montreal, Quebec, Canada.

**Keywords:** deliberate practice, end of life, fellowship education, focus group, palliative care education, pediatrics

## Abstract

**Background:**

Simulations are an important modality for practicing high-acuity, low-frequency events. We implemented a deliberate practice simulation-based workshop to improve pediatric end-of-life care skills (PECS) competence.

**Purpose:**

To understand pediatric subspecialty fellows' perceptions about influences of a simulation-based workshop on PECS provided at the bedside several months following participation.

**Methods:**

Pediatric subspecialty fellows were recruited to voluntary focus groups during regular educational sessions six months following PECS workshop participation with aims to identify perceptions about their workshop participation and any implication on their clinical practice. Inductive qualitative content analysis of focus group interview data was performed adhering to the Standards for Reporting Qualitative Research.

**Results:**

Ten fellows participated in one of three focus groups. Researchers identified three major themes of fellow experience: burden, safe practice space, and self-efficacy. Fellows described practice implications from workshop participation, including incorporation of specific practices, improved anticipatory guidance, and increased team leader confidence.

**Conclusions:**

Targeted, deliberate simulation-based practice of PECS can help close the gap from learning to practice, contributing to provider self-efficacy and potentially improving clinical care for pediatric patients and families at end of life.

## Introduction

Simulation is a well-established method for teaching and learning communication skills, including for end-of-life (EOL) care education.^1–10^ Most findings report increased learner efficacy immediately following simulation sessions,^1,9–18^ yet fewer studies explore the learning efficacy weeks to months following participation. The purpose of this study is to understand pediatric subspecialty fellows' participants' perceptions about influences of a simulation-based workshop on pediatric end-of-life care skills (PECS) provided at the bedside several months following participation.

Simulation education seeks to create safe learning spaces where health care professionals can practice and refine a broad range of skills.^1^ Participants can gain experience with challenging situations not encountered regularly in clinical practice or too high stakes to practice clinically without prior education.^2^

As noted by the American Academy of Pediatrics, pediatric death is a rare part of pediatric practice,^3^ and simulation can effectively address these situations for pediatric trainees who lack significant exposure to EOL care during medical training.^4^ Pediatric residents have reported improved perceptions of their communication skills at EOL following an educational intervention involving simulation focused on providing EOL care.^4^ One study found mentor observation and personal experience to be the most valuable tools in helping clinicians care for a child at the EOL.^5^ This takes time, experience, and a quality mentor-mentee relationship, however, plus the situations must spontaneously arise when mentor and mentee are both in the clinical learning environment.

Many groups have implemented palliative care simulation workshops and courses to enhance provider and interprofessional team members' abilities at all levels, from students to practicing clinicians.^6–10^ Compared to didactic courses alone, learning sessions that include simulation are an effective method for improving learner comfort in providing palliative and EOL care.^6^ One group showed that participants' simulation sessions improved their knowledge and self-awareness on post-session tests and quality performance, as measured by direct observation with a competency evaluation.^7^

Focused PECS education can teach EOL communication skills,^8^ and new clinicians report increased knowledge about available supports, as well as improved listening skills, support for families, and provision of medications at EOL.^9^ Pediatric critical care fellows reported an increase in both preparedness for difficult conversations and confidence in communication surrounding EOL care with simulation-based training specific to EOL communication.^10^ Their group recommended EOL care teaching as a standard part of pediatric critical care training.^10^ Overall, participants at multiple levels perceive an improvement in their comfort and skills following simulation participation and intentional PECS education.

Many of the studies published provide survey data from before, during, and after participation in an EOL simulation, and participants generally perceive training as beneficial.^1,9–18^ Some studies elicited detailed feedback on the perceived benefits of the simulation experience in the form of focus groups^9,13^ or guided reflections with the participants.^8^ Other studies focused primarily on giving participants feedback on their performance.^19,20^ While all of these outcomes are important toward program evaluation, there is a paucity of reporting on higher level outcomes, including participant behaviors and any clinical impact outside the workshop, as conceptualized by Kirkpatrick.^7^ Overall, there is an educational gap in understanding participant perceptions about ongoing practice implications following pediatric EOL workshops.

Our group conducts annual workshops in PECS for subspecialty fellows across several subspecialties (neonatal-perinatal medicine, pediatric critical care, hematology oncology, cardiology, pulmonology, blood and marrow transplant, nephrology, and hospital medicine). Fellows are required to attend a half-day workshop once or twice during their training. Workshops were developed with a deliberate practice model and include the following:

- didactic content;
- simulated communication scenarios with standardized patient parents;
- skills stations for communication, symptom management, and EOL extubation.

Immediate formative feedback and debriefing with interprofessional facilitators are emphasized across all portions. The workshop is feasible and effective as assessed by a survey of participant self-efficacy immediately following the workshop.^16^ For this study, we aimed to understand pediatric subspecialty fellows' perceptions about influences of the PECS simulation-based workshop on PECS provided at the bedside several months following participation through focus groups.

## Methods

This study was conducted and reported according to the Standards for Reporting Qualitative Research.^21^ Researchers and expert practice educators at a Midwest academic hospital implemented a workshop in 2018 for pediatric subspecialty fellows with a goal to improve competence in care at EOL for pediatric patients and their families. Researchers are doctorally prepared and have conducted numerous qualitative studies. Expert practice educators have been serving in academic and clinical medicine for over eight years. In this half-day workshop, participants learned and practiced PECS through a combination of didactic instruction and immersive simulation.^16^

To address our study aim, we used focus group methodology to stimulate participant interactions, explore transfer of PECS workshop knowledge, skills, and attitudes, and draw out self-awareness perceptions of practice performance at the bedside at the EOL in experiences with pediatric patients and their families.^7,22^ A purposive, convenience sample was recruited from the original groups of EOL workshop participants.

We intentionally invited neonatal-perinatal medicine, pediatric hematology and oncology, and critical care fellows who had participated in one of the workshops (*n* = 16) with the goal of obtaining a sample of at least two participants from each subspecialty and two from each year of training. We did not attempt to reach sampling saturation; instead, we intentionally sought participant experiences across groups. Participation was voluntary, and the institutional review board deemed the study exempt from full review. In the spring of 2019, 16 pediatric fellows were invited to participate in one of three 90-minute focus groups.

We utilized focus groups instead of a survey or interviews to capitalize on perceptions shared within conversations and interactions between participants.^23^ An interview guide for the focus group was developed to access emotive dialog, explore practice examples, and derive meaningful responses (Table 1).^22^ Approximately six months following participation in the workshop, and in the week before meeting together, researchers sent an introductory letter through email, including the five guiding focus group questions (Table 1). Facilitators (H.K. and J.M.S.) led focus groups during regularly scheduled 90-minute subspecialty educational sessions. Sessions were recorded for accuracy and transcribed for analysis. Data were de-identified and analyzed through content analysis methods.^24^

**Table 1.** Pediatric Fellow End-of-Life Care Skills Focus Group Questions

1. What is the most difficult part of caring for a patient and their family near the end of a child's life?
2. How has the EOL workshop influenced your care for patients and families at the EOL since the time of the workshop?
3. How has what you learned at the EOL workshop affected your emotional response to caring for pediatric patients at the EOL?
4. What was the most difficult part about the EOL workshop?
5. What was the most powerful lesson you took away from the EOL workshop?

EOL, end of life.

Researchers (N.G., H.K., S.M., and J.M.S.) individually read each transcript to get a sense of the whole. Using an asynchronous, modified scissor and sort technique, the data were broken up into meaning units and condensed to address the study objective. A code list of themes and subthemes was formed from the condensed meaning units,^25^ and all transcripts were re-read according to the themes and subthemes. Conflicts in coding were resolved through consensus at a synchronous group meeting. Researchers addressed study trustworthiness by creating a memoing trail of thoughts throughout the analysis, recording detailed descriptions of data as it evolved, maintaining an initial and final list of themes and subthemes, and tracking how the evolving themes and subthemes addressed the study objective for the purpose of interpretation and conclusions.^26^ Actual quotes from fellows are used to enhance the credibility of data (Table 2).

**Table 2.** End-of-Life Care Skills Trainee Perceptions: Themes, Subthemes, and Supporting Quotations

Themes	Subthemes	Supporting quotation(s)
Burden	Uncertainty	The imprecision of the process of death… Sometimes things happen way faster than I've expected them to. Sometimes they happen way slower. I think that can be hard on the family, of course, but us also, hard on me. We didn't think she would live that long. We were expecting several days to a couple weeks, and she ended up like a couple months.
	Emotion	I think there is quite an emotional burden that comes with it… as you are going through the process with the family. That's always been something that I've struggled [with]… like I'm using the family, more or less, to gain experience, and that kind of makes me feel guilty in some ways. It can be nerve-racking […] the different conversations you may have. I have a hard time knowing how best to portray my emotions to the family. Terrified Sadness Reassurance that it's okay to show your emotions to the family. Nice to have a place to fail where it's not real life staring back at you.
	Leadership	I think what's hard is you have to get a read on the room when you walk in, because every family is going to react to the whole process differently, and there's a wide variety of variables that lead to that: cultural background; educational background; prior experience. My difficulty is finding the fine line where you are trying to support and give facts to the family so they can make the best decision for the patient or their child without inducing their response. We do feel the responsibility as the doctor or the main caretaker that we kind of have to be guiding that boat and lea[d]ing everyone into a similar experience where everyone will feel comfortable with the outcome.
Safe practice space	Direct communication	… reminding myself to be very direct in my language when I'm talking to families or whomever. It's very easy, almost a protective mechanism, I think, to fall back on euphemisms or be circuitous with what you're saying, but I think that doesn't help anybody in the long run. I think for me it was helpful to go through a general idea, that there's not a wrong way or a right way, but still, it's good to know what are the preferred phrases that you should use, the preferred approach when you're going into that situation… Then you can customize or modify that approach. I could have reacted to the parents or the family for their sadness or emotions, but it shouldn't be only that part. That should be a part of it, but it should be also leading toward the goal [to address end of life planning].
	Logistics	I think the workshop just provided some, both legitimacy, and structure to the whole conversation about death and dying… It's one of those things that you don't really read about. I really try to focus on letting there be silence.
	Physiologic management	Descriptive technique [is] part of pain control, pain management. Weaning your ventilator to develop a little natural hypercarbia, and rapid escalation of your opiates is okay, and really targeting comfort over respirations.
	Appreciation for professional feedback	You can't have this conversation, pronounce the child, and then pull the family aside and be like, “Hey, so can I get some feedback on how I did with you?” You might want to communicate certain things and you don't know how people are receiving it, and it was nice to get that from it. “Oh, when you say these, I actually thought you meant these,” and “I felt this way.” That was for me valuable. It's not real and you do get some feedback and you can learn from it and you can make mistakes there. Practicing with the standardized patients and actually seeing their real-life emotional feedback and responses was reassuring. The feedback that you can get from those simulated patients. They reenact real families and it is a one-time deal.
	Informed practice	You guys provided us with that handout on dosing, of how to escalate. That was super helpful to have that. I made copies. Practicing of the actual words you were going to say was very key. [The workshop helped give] legitimacy and structure to the whole conversation about death and dying.
Self-efficacy	Self-confidence	I feel like the workshop definitely increased my confidence when talking to families… I was terrified to go in and talk to this family a couple weeks ago… It actually went OK, and I have no doubt that that was from having practiced it before. It is really just, I think, confidence building, because I had not, at least to that point, run, or at least been the lead on, one of these conversations, and literally, it was like the week afterward that I led a discussion with a family on end of life, so I think it was really just confidence and being able to practice beforehand.
	Normalcy of discomfort	Kind of the normalcy of discomfort, I guess, and how that doesn't necessarily get better over time. It's always going to be a little bit of a hard situation, so the validation of that. It's not supposed to be easy.
	Flexibility	I feel like I now am equipped to go to a situation where I might have less support and be able to still cover those bases and think about things that I maybe previously before the workshop wasn't even realizing were happening. You sometimes get questions that you don't know, and being able to gracefully answer that in a high-stakes situation and say, “I don't have that answer, but I'll get that for you.” I used to always think there was one specific way of having these discussions, but doing the workshop… I realize that there's a multitude of ways of doing it and they're not wrong.

## Results

Ten fellows participated in one of the three focus groups. All programs invited (neonatal-perinatal medicine, pediatric hematology and oncology, and critical care) and years (post-graduate years one through three) were represented.

Researchers identified three major themes of pediatric fellow perceptions of the influence of the EOL workshop experience on subsequent EOL care for dying children and their families: the burden of EOL care, safe practice space, and self-efficacy. Subthemes and supporting exemplar quotations detail perceptions that emerged and include the following:

- Theme burden, subtheme uncertainty, “The imprecision of the process of death… Sometimes things happen way faster than I've expected them to. Sometimes they happen way slower. I think that can be hard on the family, of course, but us also, hard on me.”
- Theme burden, subtheme leadership, “We do feel the responsibility as the doctor or the main caretaker that we kind of have to be guiding that boat and lea[d]ing everyone into a similar experience where everyone will feel comfortable with the outcome.”
- Theme safe practice space, subtheme appreciation for professional feedback, “You can't have this conversation, pronounce the child [dead], and then pull the family aside and be like, ‘Hey, so can I get some feedback on how I did with you?’”
- Theme self-efficacy, subtheme self-confidence, “I feel like the workshop definitely increased my confidence when talking to families… I was terrified to go in and talk to this family a couple weeks ago… It actually went OK, and I have no doubt that that was from having practiced it before.”
- Theme self-efficacy, subtheme flexibility, “I used to always think there was one specific way of having these discussions, but doing the workshop… I realize that there's a multitude of ways of doing it and they're not wrong.”

Table 2 contains all sub-themes and their exemplar quotations.

In all focus groups, fellows described translating specific skills gained from the EOL workshop to the bedside with patients and families. Real-world practice implications that pediatric fellows identified stemming from their workshop participation are further described in Table 3. These skills included increased confidence as a leader, improved anticipatory guidance, and incorporation of skills specifically learned and practiced during the workshop.

**Table 3.** Workshop Informed End-of-Life Care Practice Skills

Increased confidence leading and directing EOL conversations
Improved ability to guide conversations with families to goals of care
Ability to provide anticipatory guidance regarding the dying process with care team and family
Using direct language and focusing on clear communication with families
Being cognizant of emotional matching between provider and family
Ensuring time for silence
Having a “toolbox” of helpful words and phrases to use during EOL conversations

## Discussion

We aimed to understand the pediatric subspecialty fellows' perceptions about influences of a simulation-based workshop on PECS provided at the bedside several months following participation. Our findings demonstrate that subspecialty pediatric fellows perceive the EOL workshop to be a safe space to practice EOL communication and skills. Across the six months following deliberate practice in the simulation-based PECS workshops, fellows reported incorporating learnings into active clinical practice. Our findings suggest perceptions of long-lasting skill development translated to real-world practice changes during the six months after the workshop. These findings begin to fill the gap in the literature regarding potential long-term outcomes following simulation-based EOL education.

Participants reported implementation of several strategies learned in the PECS workshop in distressing clinical scenarios. Strategies included using direct language, using preferred phrases, and knowing how to structure a difficult conversation. Participants also reported perceptions of freedom in conducting difficult EOL conversations after learning that there are multiple reasonable and correct ways to do so. Fellows reported an increase in confidence in having conversations with families regarding EOL, despite having to do so while experiencing continued emotional distress.

Our study has some limitations. First, our study relied on trainee self-report; there was no direct observation by supervisors or interprofessional team members on how they incorporated skills nor their competency in this area. Findings are limited to outcomes for participants at a single institution that provided one method of simulation-based PECS education. Finally, findings are limited to a single time period and are not longitudinal.

In our study, self-report by these participants provided important themes, subthemes, and practice implications. We demonstrate lasting clinical implications following this simulation-based educational intervention for these participants. Future work should consider how to gather data on even longer-term practice influence of PECS education and to reach data saturation. Focus groups with participants from additional workshops and specialties, as well as a longitudinal approach might be considered.

Finally, subspecialty fellows provide care for patients and families along with multiple interprofessional team members. As the local workshop expands to include other health care team members, it will be imperative to collect their perceptions on lasting clinical practice implications of the workshop. It will also be important to consider direct observation of PECS competence by supervisors, interprofessional team members, and bereaved parents.

In conclusion, targeted, deliberate simulation-based practice of PECS can help close the gap from learning to practice, contributing to provider self-efficacy and potentially improving clinical care for pediatric patients and families at EOL.

## Abbreviations Used

EOL: end of life
PECS: pediatric end-of-life care skills
